# Comparison of benign peritoneal fluid- and ovarian cancer ascites-derived extracellular vesicle RNA biomarkers

**DOI:** 10.1186/s13048-018-0391-2

**Published:** 2018-03-02

**Authors:** Cindy M. Yamamoto, Melanie L. Oakes, Taku Murakami, Michael G. Muto, Ross S. Berkowitz, Shu-Wing Ng

**Affiliations:** 1 Hitachi Chemical Co. America, Ltd. R and D Center, 1003 Health Sciences Rd, Irvine, CA 92617 USA; 2 Department of Obstetrics, Gynecology and Reproductive Biology, Gynecologic Oncology Division, Brigham and Women’s Hospital, Harvard Medical School, 75 Francis Street, Boston, MA 02115 USA; 3 Department of Obstetrics and Gynecology, Tuft Medical Center, 800 Washington Street, Boston, MA 02111 USA

**Keywords:** Extracellular vesicles, Ovarian cancer, Biomarkers, Ascites, Peritoneal fluids

## Abstract

**Background:**

Extracellular vesicles (EVs) are considered as a new class of resources for potential biomarkers. We analyzed expression of specific mRNA and miRNA in EVs derived from ovarian cancer ascites and the ideal controls, peritoneal fluids from benign patients for potential early detection and prognostic biomarkers.

**Methods:**

Fluids were collected from subjects with benign cysts or endometrioma (*n* = 10), or low/high grade serous ovarian carcinoma (*n* = 8). EV particles were captured using primarily ExoComplete filterplate or ultracentrifugation and analyzed by nanoparticle tracking analysis, ELISA, and scanning electron microscopy. EV RNAs extracted from two ascites and three peritoneal fluids were submitted for next-generation sequencing. The expression of 34 mRNA and 18 miRNAs in the EVs isolated from patient fluids and cell line media was determined using qPCR.

**Results:**

EVs isolated from patient samples had concentrations greater than 10^10^ EV particles/mL and 30% were EpCAM-positive based on ELISA. EV particle sizes averaged 113 ± 11.5 nm. The qPCR studies identified five mRNA (*CA11, MEDAG, LAMA4, SPINT2, NANOG*) and six miRNA (*let-7b, miR23b, miR29a, miR30d, miR205, miR720*) that were significantly differentially expressed between cancer ascites and peritoneal fluids. In addition, *CA11* mRNA was decreased to 0.5-fold and *SPINT2* and *NANOG* mRNA were significantly increased up to 100-fold in conditioned media of cancer cells compared to immortalized ovarian surface and fallopian tube epithelial cell lines, the hypothesized cells of origin for ovarian cancer development.

**Conclusions:**

This study indicates that EV mRNA profiles can reflect the disease stage and may provide a potentially novel source for discovery of biomarkers in ovarian cancer.

**Electronic supplementary material:**

The online version of this article (10.1186/s13048-018-0391-2) contains supplementary material, which is available to authorized users.

## Background

Ovarian cancer is the fifth-leading cause of cancer deaths in women [1]. With a lack of early obvious symptoms, women are frequently diagnosed with advanced stage disease. Approximately 60% of women are diagnosed at stage 3 or higher where the 5-year survival rate is below 30% [2]. In contrast, only 15% of cases are diagnosed at stage 1, when the tumor is localized to the primary site and patients have a 5-year survival rate of about 92%. In addition, patients with metastatic ovarian cancer frequently experience high recurrence rates within 16–22 months after conventional platinum-based combination chemotherapy. Identification of novel, specific and sensitive biomarkers for screening, monitoring or prediction may improve clinical outcomes and survival.

Screening tests currently used for ovarian cancer detection include pelvic examination, transvaginal ultrasound, and cancer antigen 125 (CA125). If an adnexal mass is detected by physical examination and/or ultrasound, surgery is ultimately needed for the confirmed ovarian cancer diagnosis and staging. CA125 is more routinely used as a marker for disease recurrence and treatment response [3]. It has been shown to be elevated in 80% of epithelial ovarian carcinomas, but its increase in other conditions such as endometrial, pancreatic and breast cancer and certain benign conditions have limited its use as an early screening marker [4, 5]. In addition, annual screening with both CA125 and transvaginal ultrasound has not reduced ovarian cancer mortality compared with usual care [6]. Further research is necessary to discover and identify biomarkers that would be effective in early ovarian cancer screening.

Recently, extracellular vesicles (EVs) have been analyzed for their potential as ovarian cancer disease biomarkers [7]. EVs comprise of exosomes (30–100 nm) and microvesicles (100–1000 nm) which are either actively released from cells by fusion of multivesicular bodies to plasma membrane or formed by direct budding of the cell membrane into the extracellular space, respectively. Exosomes and microvesicles contain proteins, lipids and nucleic acids such as mRNA and miRNA from their cell of origin. The increasing evidence of their roles in cell-to-cell communication [8, 9], their high abundance in plasma (10^12^ per mL), and highly stable nature, are several of the reasons for the increased interest to identify EV-based biomarkers. In ovarian cancer, EVs have been explored in ascites and urine for miRNA and protein surface markers [10, 11]. Because of the demonstrated roles of EVs in communication and tumor progression, we initiated a study to examine the expression of EV mRNA and miRNA in ovarian cancer ascites and compared the expression with an ideal but difficult to obtain control source, peritoneal fluids from females inflicted with benign gynecologic diseases. These studies indicate that malignant ascites EVs package quantifiable mRNA and miRNA that can potentially provide insights into diagnostic biomarkers and therapeutic targets.

## Methods

### Study design

The experimental design is separated into two parts: 1) preliminary characterization of clinical samples and 2) evaluations of the biomarkers in cultured cell line. For the characterization of clinical samples, four main studies were performed: 1) size and concentration of EVs, 2) preliminary qPCR screening of mRNA biomarkers identified through previous literature, 3) pilot RNA-sequencing of EV mRNA, and 4) RT-qPCR validation of mRNA and screening of miRNA. The mRNA biomarkers identified through the RNA-seq qPCR validation were evaluated in several appropriate cell lines.

### Biofluid collection

Ascites from advanced stage ovarian cancer patients were collected from the peritoneal cavity using Yankauer suction connected to a drainage bag, or bulb suction from volumes smaller than 500 mL. Bulb suction was also used to collect small volumes (1–5 mL) of peritoneal fluids from patients with non-malignant conditions. Samples were collected at the Brigham and Women’s hospital under informed consent and Internal Review Board approval. Fluids were transferred into 50 cm^3^ tubes and centrifuged at 2000 x g for 15 min at 4 °C to remove cell debris. The supernatant was then stored at − 80 °C until further use.

### Cell culture

Immortalized human fallopian tube secretory epithelial cell line (FTSEC) was kindly provided by Dr. Ronny Drapkin [12]. Ovarian surface epithelial (OSE7, HOSE1–15) and high-grade serous ovarian cancer (SKOV3, OVCA3) cell lines have been previously described [13]. FTSEC cells were cultured in DMEM/Ham’s F-12 1:1 (Cellgro, Mediatech, Inc. Manassas, VA) supplemented with 2% Ultroser G serum substitute (Pall Corp., Port Washington, NY). Ovarian epithelial cell lines were cultured in a mixture of medium 199 and MCDB105 medium (1:1) (Sigma, St. Louis, MO) supplemented with 10% fetal bovine serum (FBS, Invitrogen, Carlsbad, CA).

Standard media was replaced with exosome-free fetal bovine serum (System Biosciences, SBI, Palo Alto, CA) containing media 24 h prior to conditioned media collection. Cell counts were determined at the time of conditioned media collection and ranged from 2 to 9 × 10^5^ cells/mL. Conditioned media were transferred to 50 cm^3^ tubes and centrifuged at 2000×g for 15 min at 4 °C to remove cells and cell debris. The supernatant was then stored at − 80 °C until further use.

### Differential ultracentrifugation and EV characterization

Ascites and peritoneal fluid samples were initially centrifuged at 2000 x g for 10 min to remove large debris. The supernatant was collected and further centrifuged at 10,000 x g for 30 min. EVs in the supernatant were then collected by ultracentrifugation at 100,000 x g for 1 h, washed with PBS, then collected again at 100,000 x g for 1 h in a Ti90 rotor. EV pellet was resuspended in PBS and stored at − 80 °C until further use. Nanoparticle tracking analysis of the EVs was conducted by Nanosight LM10 (Particle Characterization Laboratories, Inc., Novato, CA). Samples were diluted from 1:5 to 1:50 and applied to ELISA assay for EpCAM detection (Thermo Scientific, Frederick, MD) at 450 nm.

### Scanning electron microscopy (SEM)

Ascites samples were pre-centrifuged at 3000 x g for 15 min at 4 °C before applying to the ExoComplete filterplate (Hitachi Chemical Diagnostics, Inc., (HCD), Mountain View, CA). EVs were captured on the filter membrane after centrifugation at 2000 x g for 5 min. Filters were fixed with 4% paraformaldehyde, and blocked with casein before incubation with 1:200 anti-human CD63 (Clone H5C6, BioLegend, Dedham, MA) for 1 h with gentle shaking. Samples were washed 3 times with casein PBS followed by incubation with 1:40 goat anti-mouse IgG gold colloid 9.0–11.0 nm (Sigma-Aldrich, St. Louis, MO) for 2 h. EVs were washed 3 times with casein PBS and PBS. Samples were fixed again with 4% paraformaldehyde for 5 min. Samples were washed once with PBS and 2 times with distilled water before incubation with 100 μL Silver Enhancement solution (BBI Solutions) for 10 min. Filters were washed with distilled water and air-dried overnight before analysis with SEM using Hitachi S-4800.

### Next generation RNA sequencing

Ascites (*n* = 2) and peritoneal fluid (*n* = 3) samples were centrifuged at 2000 x g for 10 min at 4 °C after thawing as described above. The ExoComplete filterplate (HCD) captured EVs from 400 μL of centrifuged ascites and peritoneal fluid supernatant. Total RNA from EVs attached to the filter membrane was isolated using miRNeasy kit (Qiagen, Valencia, CA). Total RNA was monitored for quality control using the Agilent Bioanalyzer Nano RNA chip and Nanodrop absorbance ratios for 260/280 nm and 260/230 nm. Library construction was performed according to the Illumina TruSeq mRNA stranded protocol. The input quantity for total RNA was within the recommended range and mRNAs and noncoding RNAs with poly(A) tails was enriched using oligo dT magnetic beads. The enriched poly(A)^+^ RNA was chemically fragmented. First strand synthesis used random primers and reverse transcriptase to make cDNA. After second strand synthesis, the ds cDNA was cleaned using AMPure XP beads, cDNA was end repaired and then the 3′ ends were adenylated. Illumina barcoded adapters were ligated on the ends and the adapter ligated fragments were enriched by nine cycles of PCR. The resulting libraries were validated by qPCR and sized by Agilent Bioanalyzer DNA high sensitivity chip. Concentrations for the libraries were normalized and then multiplexed together. Multiplexed libraries were sequenced using paired end 100 cycles chemistry for the HiSeq 2500. The version of HiSeq control software was HCS 2.2.58 with real time analysis software, RTA 1.18.64. FASTQ files were input into Maverix Biomics platform mRNA-seq for differential expression in eukaryotes version 2.5. Additionally, Ingenuity Pathway Analysis (IPA) (Qiagen) was employed to identify biological pathway modulation.

### Extracellular vesicle mRNA analysis

Ascites (*n* = 8), peritoneal fluid (*n* = 10), and cell culture conditioned media (2 mL) were processed by first thawing for 10 min at 37 °C and then placed on ice. For preliminary qPCR screening of mRNA in clinical samples, ascites (*n* = 8) and peritoneal fluid (*n* = 2) were used. Thawed samples were centrifuged at 2000 x g for 10 min at 4 °C. Three hundred fifty μL of supernatant was applied to ExoComplete filterplate (HCD) and centrifuged. For cell culture conditioned media, samples were centrifuged as above and supernatants were applied to EV collection tubes (HCD). EVs were then captured onto the filter membrane after repeated centrifugation. From this step, procedures for mRNA analysis for ascites, peritoneal fluid, and cell culture conditioned media are identical. Exocomplete lysis buffer is applied to the EVs captured on the filter and incubated at 37 °C for 10 min. Centrifugation of the filterplate or filter tips from the collection tube was performed at 2000 x g for 5 min at 4 °C to transfer lysate to the mRNA Capture Plate for hybridization of mRNA to the oligo(dT)-covalently linked wells. After wash steps, on-plate random-primed cDNA synthesis using MMLV was performed at 37 °C for 2 h. For qPCR analysis, 2 μL of cDNA was used with Sso Advanced SYBR mix (Bio-Rad, Hercules, CA) and gene-specific primers (Additional file 1). Real-time PCR was performed on a ViiA7 (Thermo Fisher Scientific, Inc., Waltham, MA) instrument using the following profile: initial denaturation at 95 °C for 10 min, 40 cycles of 95 °C for 30 s and 65 °C for 1 min, melting curve analysis. Ct values greater than 36 were set to 36 cycles for data analysis. Real-time PCR data was processed by Data Assist v3.01 (Thermo Fisher Scientific, Inc.) and analyzed by Excel.

### Extracellular vesicle miRNA analysis

Ascites (*n* = 8) and peritoneal fluid (*n* = 10) were thawed for 10 min at 37 °C and then placed on ice. Four hundred μL ascites and peritoneal fluid were centrifuged at 2000 x g for 10 min at 4 °C. Supernatant was applied to ExoComplete filterplate and centrifuged at 2000 x g for 5 min at 4 °C. Lysis buffer from miRNeasy (Qiagen), was applied to the wells in the filterplate. Total RNA was isolated per manufacturer’s protocol. Synthesis of cDNA was performed using miScript RT kit (Qiagen). The cDNA was diluted 1:4 and 1 μL was used with the miScript PCR assay (Qiagen) for qPCR screening. For miRNA analysis, Excel and DataAssist v3.01 was used with the following parameters: cut-off value of Ct = 36, *SNORD61* selected as endogenous reference RNA.

### Statistical analysis

The statistical analysis was performed using Microsoft Excel software. The statistical significance of the differences was determined by applying the Student’s *t*-test.

## Results

### Patient characteristics and samples

The patient characteristics and clinical information were obtained for ovarian cancer ascites samples (Additional file 2). All samples were collected at the time of diagnosis. Eight patients were diagnosed with serous type ovarian cancer: seven with high grade, and a single patient was diagnosed with low grade serous type. The ages ranged from 48 to 80 years with an average age at diagnosis of 64 ± 12 years old. Using the available data, CA125 levels were elevated with average values at 1289 ± 541 U/mL at diagnosis and average progression-free and overall survival were 19 ± 8 and 33 ± 22 months, respectively. For peritoneal fluid samples, no malignant cells were identified in peritoneal washings or from ovary, fallopian tube, uterus and cervix pathology report (Additional file 3).

### Ascites and peritoneal fluid EV characterization

**Fig. 1:**
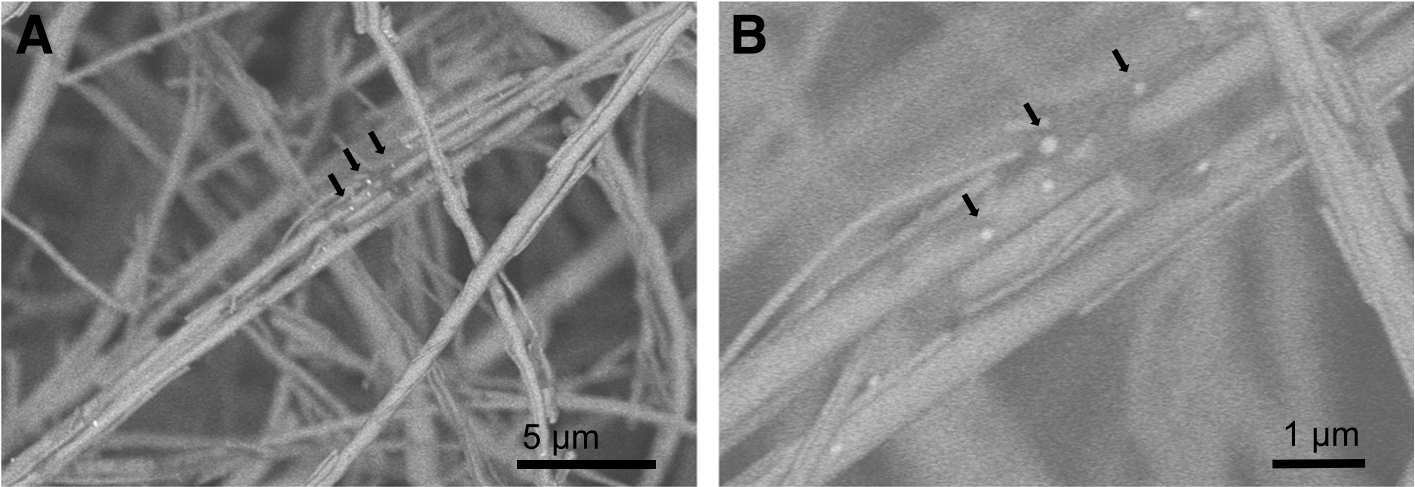
**a**,**b** Scanning electron microscopy of ovarian cancer ascites EVs captured on the membrane of the ExoComplete filterplate. Ovarian cancer ascites was applied to the filterplate, fixed, labeled with anti-CD63 primary monoclonal antibody and anti-mouse IgG colloidal gold with silver enhancement. Black arrows indicate selected extracellular vesicles with diameters < 200 nm captured on the membrane. Images were obtained with accelerating voltages of 2.0 kV in detection mode

Ascites and peritoneal fluid extracellular vesicles were isolated using differential ultracentrifugation and characterized for size and concentration using nanoparticle tracking analysis (Table 1). This preliminary characterization indicated average sizes of ascites and peritoneal fluid EVs were not statistically different and averaged 113 ± 11.5 nm. CD63-positive particles < 200 nm in diameter were observed in scanning electron micrographs of ovarian cancer ascites samples applied to the ExoComplete EV capture filterplate and correlate to size estimates from nanoparticle tracking analysis (Fig. 1). EV concentrations ranged from 10^10^ to 10^12^particles/mL and were also not statistically significant between the two sample types. EVs from both ascites and peritoneal fluids were also evaluated for EpCAM surface marker expression by ELISA. EpCAM is a proposed surface marker of ovarian cancer-derived exosomes. Only one of each sample type was found to be positive for EpCAM. The single ascites and peritoneal fluid-derived EV samples had an EpCAM concentration of 670 pg/mL and 1.44 ng/mL, respectively. The other samples were below the limit of detection (50 pg/mL) for the ELISA assay.

**Table 1:** Ascites (*n* = 3) and peritoneal fluid (*n* = 3) EV characteristics

EV Sample Source^a^	ID	EpCAM ELISA^a^	Concentration (particles/mL)	Size (nm)
Ascites	A2	positive	2.17E + 10	105
Ascites	A3	negative	3.20E + 12	106
Ascites	A10	negative	5.43E + 10	128
Peritoneal Fluid	B7	negative	1.20E + 10	106
Peritoneal Fluid	B11	positive	8.47E + 11	106
Peritoneal Fluid	B13	negative	1.55E + 10	128

^a^EV isolated by differential centrifugation

^b^10E9 – 10E11 EVs applied

### Next generation RNA sequencing and qPCR validation

A literature search for potential ovarian cancer biomarkers identified 50 mRNA candidates (Additional file 4) which were then evaluated in a preliminary qPCR screening of ovarian cancer ascites (*n* = 8) and peritoneal fluid EVs (*n* = 2). Three mRNAs, *NANOG, SPINT2, ZEB2*, were found to be significantly elevated (> 2-fold, *p*-value < 0.05) in ascites in the preliminary screening (Additional file 5). To further identify differentially expressed genes and additional mRNA markers, next generation poly(A)^+^ RNA sequencing was performed on selected samples of ascites (*n* = 2) and peritoneal fluid (*n* = 3). RNA sequencing mapping percentages to hg19 assembly ranged from 50% to 87%, and greater than 70% of the reads were kept after quality assessment (Additional file 6). The read distributions indicate that both samples have a higher percentage of reads mapping to exons and 3’ UTR compared to introns and 5’ UTR as expected based on sequencing preparation methodology (Additional file 7). Intergenic regions ranged from 2.5–15.9% for peritoneal fluid EVs and 24–47% for ovarian cancer ascites EVs.

Pathway analysis of RNA-seq differential gene expression data from ovarian cancer ascites and peritoneal fluid EVs identified organismal injury, cancer and reproductive system diseases as the main categories of diseases and disorders (Additional file 8). In terms of function, results were consistent with the biological environment of an advanced stage disease. Signaling pathways within growth, malignant tumor and advanced malignancy categories were predicted to be up-regulated whereas tumor cell death pathways were predicted to decline.

**Fig. 2:**
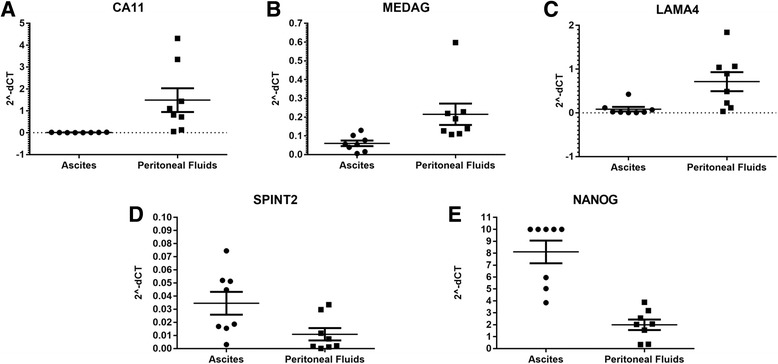
Relative gene expression in peritoneal fluids (*n* = 10) and ovarian cancer ascites (*n* = 8) EV samples. **a**-**e** *CA11, MEDAG, LAMA4, SPINT2, NANOG* normalized to *ACTB* are shown as 2^-ΔCT gene expression levels with average and SD indicated by horizontal lines. Maximum gene expression was set at a cut-off of 10. Gene expression values for each individual subject are represented as solid circle and square symbols for ascites and peritoneal fluids, respectively. Statistical significance for (**a**-**d**) is *p* < 0.05 and (**e**) is *p* < 0.005 using Student’s t-test

RNA-seq differential gene expression analysis identified 114 genes with statistical significance (*p* < 0.05) (Additional file 9). From this list, 30 genes selected for qPCR validation based on fold change, *p*-value, abundance and function were measured in eight ovarian cancer ascites and ten peritoneal fluid samples (Additional file 10). *SPINT2*, one of the three mRNA found to be differentially expressed in the initial screen based on literature-identified biomarkers was identified in the RNA-seq analysis. The remaining two genes, *NANOG* and *ZEB2*, were added to the list of 30 genes in the final RNA-seq qPCR validation assays*. ACTB* was selected as a reference gene for mRNA normalization. Of the selected mRNA for qPCR validation, five were found to be significantly (*p* < 0.05) differentially expressed in ovarian cancer ascites and peritoneal fluid (Fig. 2). Three mRNAs, *CA11, LAMA4, MEDAG*, were .01–.28-fold lower expressed and two mRNA, *SPINT2* and *NANOG*, were 3.2–5.8-fold higher expressed in ovarian cancer ascites versus peritoneal fluid EVs.

### Ascites and peritoneal fluid EV miRNA analysis

**Fig. 3:**
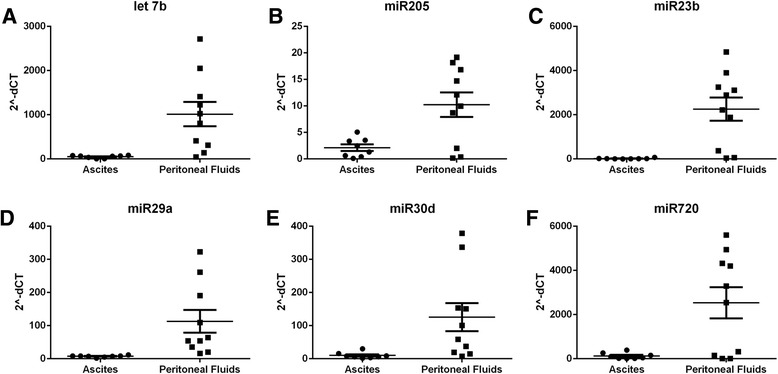
Relative miRNA expression levels in peritoneal fluids (*n* = 10) and ovarian cancer ascites (*n* = 8) EV samples. **a**-**f** *Let7b, miR205, miR23b, miR29a, miR30d, miR720* normalized to *SNORD61* are shown as 2^-ΔCT expression levels with average and SD indicated by horizontal lines. Gene expression values for each individual subject are represented as solid circle and square symbols for ascites and peritoneal fluids, respectively. Statistical significance for (**a**-**d**) is *p* < 0.05 and (**e**) is *p* < 0.005 using Student’s t-test

Small RNAs are abundant within extracellular vesicles. Here, we quantitate specific miRNA previously identified to be involved in ovarian cancer progression or invasiveness [14–18]. There were six miRNAs, *let-7b, miR23b, miR29a, miR30d, miR205, miR720*, which were found to be significantly (p < 0.05) decreased .01–.21-fold in ovarian cancer ascites compared to benign peritoneal fluid when normalized to *SNORD61* (Fig. 3).

### Multivariate discriminate analysis

The combined mRNA and miRNA raw qPCR data were used in multivariate discriminate analysis (Additional file 11). The predictors*, LAMA4, CA11, MEDAG, NANOG, SPINT2, let-7b, miR23b, and miR29a* were found to be sufficient to classify 87.5% and 100% of ovarian cancer (*n* = 8) and disease control (*n* = 10) groups, respectively.

### Specific EV mRNA from normal and cancer cell lines

**Fig. 4:**
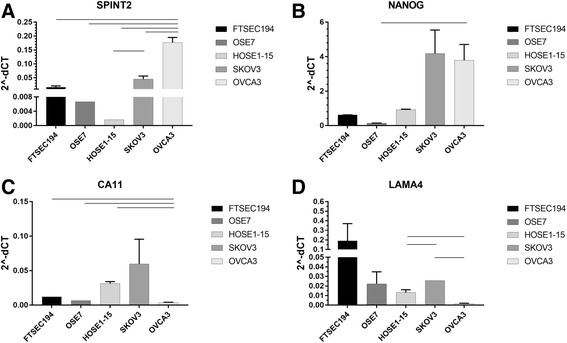
Specific mRNA expression normalized to *ACTB* from immortalized fallopian (FTSEC194), ovarian surface epithelial (OSE7, HOSE1–15) and ovarian cancer cell lines (SKOV3, OVCA3) EVs released in conditioned media. **a** *SPINT2*, (**b**) *NANOG*, (**c**) *CA11*, (**d**) *LAMA4* mRNA quantitation (2^-ΔCT) is shown as column graph with average and SD (*n* = 2) and statistical significance indicated by a bar representing *p* < 0.05 by Student’s t-test

The EVs released from normal human fallopian tube epithelial (FTSEC194), ovarian surface epithelial (OSE7, HOSE1–15), and high grade serous ovarian cancer (SKOV3, OVCA3) cell lines were analyzed for specific mRNA that were differentially expressed in ovarian cancer ascites and peritoneal fluids (Fig. 4). The immortalized normal fallopian and ovarian surface epithelial cells were used as controls. Using 3 mL of conditioned media from each cell line, we confirm that *CA11* mRNA is 0.1–0.5-fold lower abundance and *NANOG* is 50-fold higher abundance in EVs released from high-grade serous ovarian cancer cells, OVCA3*.* Although *CA11* expression appeared to be elevated in SKOV3 cells, mRNA levels for *CA11* as well as *NANOG* were not statistically significant compared to normal cells. *SPINT2*, however, was found to be significantly elevated in both SKOV3 and OVCA 3 cells compared to normal cells. *LAMA4* was expressed in 0.1-fold lower abundance in OVCA3, but up to 2-fold higher abundance in EVs originating from SKOV3 cells compared to ovarian surface epithelial cell-derived EVs (HOSE1–15). The two high grade serous ovarian cancer cell lines, SKOV3 and OVCA3, demonstrated similar expression patterns for *SPINT2* and *NANOG*, but distinct relative expression for *CA11* and *LAMA4*. *MEDAG* mRNA was present in very low levels and was not reproducibly detected in all cell lines.

## Discussion

Extracellular vesicles (EVs), including exosomes and microvesicles, are small membranous particles released from all cells and found in many biofluids. These vesicles are thought to provide a mode of cellular communication and deliver their cargo of protein, DNA and RNA, to target cells [10, 11]. As a result, EVs have been a novel source of biomarkers in a wide range of diseases. In this study, we analyzed EV mRNA and miRNA from ovarian cancer ascites and benign peritoneal fluids to determine if they can provide biological insight into metastasis and be a potential source of novel diagnostic biomarkers.

The average size and concentrations of EVs isolated from ovarian cancer ascites and peritoneal fluids were < 120 nm and at least 10^10^ particles/mL and are within range of EVs isolated from other biofluids such as plasma and urine. The variability in particle concentration observed between samples is consistent with what has been observed in previous literature [11]. In addition, EpCAM has been proposed as a marker for ovarian cancer-derived exosomes [11], but only 1 out of 3 of the ovarian cancer and peritoneal fluid EV samples were positive for EpCAM. This may be reflective of the typically low presence of adenocarcinoma cells (< 0.1%) within ovarian cancer ascites and the presence of endometrial epithelial cells in peritoneal fluids [19, 20]. The ovarian cancer ascites EV sample did not demonstrate a higher EpCAM concentration compared to the peritoneal fluid EV sample.

The ovarian cancer ascites and peritoneal fluid EVs were analyzed for differential mRNA expression. The mRNAs in this study were selected based on a combination of next generation sequencing (NGS) results and previous literature. The EV characterization and RNA sequencing were pilot analyses of the EVs and identification of new targets. IPA results confirmed RNA-seq is a methodology which may identify relevant biomarkers for diagnosis. The qPCR validation of the RNA-seq results employed more samples to confirm the gene expression pattern. Based on these qPCR analyses, the mRNAs confirmed to be decreased in ovarian cancer ascites compared to peritoneal fluid EVs included *LAMA4, CA11*, and *MEDAG*. EVs from a high grade serous ovarian adenocarcinoma cell line OVCA3 also contained less abundance of *CA11* compared to controls from immortalized epithelial fallopian and ovarian cells. Recently, fallopian tube secretory epithelial cells have been proposed to be the precursor tissue for high grade serous ovarian cancer, and immortalized human fallopian tube secretory epithelial cells are being used for studying early-stage development of high grade serous ovarian cancer [21]. Based on the publicly available genotype-tissue expression (GTEx) database, *CA11* mRNA has high baseline expression in brain and medium expression in tissues such as ovary. Although no previous studies were found to link *CA11* with gynecological cancers, this mRNA was down-regulated in human gastric cancer [22]. CA11 is a member of carbonic anhydrase family known to participate in biological processes such as formation of aqueous humor, CSF and saliva. Similar to *CA11, MEDAG* was in lower abundance in ovarian cancer ascites EVs. *MEDAG* expression, however, was either at the limit of detection or undetectable in the fallopian and ovarian cell lines used in this study suggesting low baseline expression of *MEDAG* in these tissues. The GTEx database confirms this observation and shows MEDAG expressed at higher levels in visceral adipose and arterial tissues. *MEDAG* is a gene involved in processes that promote adipocyte differentiation, lipid accumulation, and glucose uptake in mature adipocytes. Because ovarian cancer cells bind preferentially to omental fat and use human omental adipocytes as an energy source, the decrease observed in ovarian cancer ascites *MEDAG* EV mRNA may reflect changes in the ascites microenvironment [23]. A previous study using microarray has also shown a lower *MEDAG* and *LAMA4* expression in ovarian cancer compared to normal epithelial cells [24]. *LAMA4* is a member of the major family of non-collagenous constituents of basement membranes. The decrease in *LAMA4* observed in malignant ascites EV and OVCA3 EV, in contrast to the increase in SKOV3 EV, are interesting and warrants further experimental studies to evaluate the relationship. Ascites EVs could be providing cell-to-cell communication through unique molecular profiles promoting cancer cell survival within the ascites milieu.

In contrast to *LAMA4, CA11*, and *MEDAG*, the 2 mRNAs, *SPINT2* and *NANOG*, were found to be increased in ovarian cancer ascites compared to peritoneal fluid EVs. *SPINT2* was also in higher abundance in OVCA3 and SKOV3 EVs compared to controls. Interestingly, although SPINT2 is a putative suppressor, we demonstrate an increase in cancer ascites [25]. Müller-Pillasch et al. also reports that *SPINT2* expression was elevated in pancreatic cancer [26]. NANOG, on the other hand, is a DNA binding homeobox transcription factor involved in embryonic stem cell proliferation, renewal and pluripotency. *NANOG* is increased in expression from normal tissue, benign, borderline, and malignant tumors of ovarian serous cystadenocarcinomas and protein is found selectively associated with high-grade ovarian serous carcinoma [27, 28]. *NANOG* mRNA was found to be significantly increased in OVCA3 EVs compared to OSE7 control. Based on previous studies relating to these mRNA, *CA11* and *MEDAG* may be involved in maintaining malignant ascites microenvironment, while *LAMA4, NANOG* and *SPINT2* activities could regulate ovarian cancer progression and metastasis.

There were six miRNA biomarkers*, let7b, miR205, miR23b, miR29a, miR30d*, and *miR720*, significantly down-regulated in ovarian cancer ascites EVs compared to peritoneal fluids. These miRNAs have previously been shown to be involved in ovarian cancer progression, invasion or metastasis. The *let-7* family regulates downstream gene targets involved in self-renewal of mesenchymal stem cells derived from human embryonic stem cells*. Let-7b* is often dysregulated in ovarian cancer and is associated with poor prognosis [29]. Recently, *miR205* was shown to be elevated in ovarian cancer tissue and associated with tumor growth and metastasis in ovarian cancer [30]. In pre-surgical plasma*, miR720* was elevated in women who had short overall survival (< 2 years) when compared to women with long overall survival (> 4 years) after their diagnosis [31]. In contrast, *miR23b* expression is lower in epithelial ovarian carcinoma and borderline tumors than in normal ovarian tissues and benign tumors consistent with the lower abundance observed in ovarian cancer ascites in this study. *MiR23b* was shown to target cyclin G1 and suppress ovarian cancer tumorigenesis and progression [14]. *MiR29a* was also shown to have tumor suppressive effects and may contribute to cisplatin resistance of ovarian cancer cells [32, 33]. *MiR30d* also functions as a suppressor of ovarian cancer progression notably by decreasing Snail expression and blocking TGF-b1-induced EMT process [34].

Malignant ascites presents in approximately 30% of women with ovarian cancer and is frequently tapped to relieve symptoms. This fluid is composed of lymphocytes, epithelial cells, and EVs, and provides clues into ascites formation and metastatic progression. Functional analysis of each specific mRNA and miRNA will be needed to determine the biological significance of their differential expression in ovarian cancer.

## Conclusions

Here, we demonstrate that EVs from ovarian cancer ascites contain distinct RNA expression signatures from benign peritoneal fluids, control samples that are rarely available to research. The two mRNA markers *SPINT2* and *NANOG* that are upregulated in cancer ascites relative to peritoneal fluids may have potential as diagnostic biomarkers. Through continued liquid biopsy investigations, an understanding of the mechanisms involved in advanced stage disease and development of chemo-resistant disease may lead to alternative therapeutic targets and improved palliative care.

## Additional files

Additional File 1: Primer sequences (5′ to 3′) for qPCR validation. (DOCX 13 kb)

Additional File 2: Clinical information from patient ascites samples. (DOCX 13 kb)

Additional File 3: Clinical information from benign peritoneal fluid (PF) sample pathology reports. (DOCX 12 kb)

Additional File 4: Preliminary set of genes with primer sequences for screening based on literature search. Additional genes (*MET, EGFR, EPCAM, CLDN3*) were quantitated using Qiagen Quantitect Primer Assays. (DOCX 13 kb)

Additional File 5: Volcano plot displays *p*-values vs. fold change of ovarian cancer ascites (*n* = 8) and peritoneal (*n* = 2) EVs. Three mRNA (*NANOG, SPINT2, ZEB2*) show values above the fold change boundary of 2 (2-fold change) and a p-value of 0.05. Plot generated using. Data Assist v3.01 software. (PPTX 58 kb)

Additional File 6: mRNA sequencing reads and percentage aligned to hg19 assembly. (DOCX 12 kb)

Additional File 7: The distribution of EV mRNA sequencing reads mapping to human genome annotations. The percentage of reads (ave. ± SD) overlapping genomic features including exons, introns, UTR, and intergenic regions are shown for peritoneal fluids (*n* = 3) and ovarian cancer ascites samples (*n* = 2). (PPTX 115 kb)

Additional File 8: Ingenuity Pathway Analysis summary of top diseases and disorders, top canonical pathways, and top molecular and cellular functions. *P*-values are calculated using the right-tailed Fisher Exact Test and number of molecules are based on Ingenuity Knowledge Base with information contained in Canonical Pathways coming from specific journal articles, review articles, textbooks and HumanCyc. (DOCX 22 kb)

Additional File 9: Differentially expressed genes from RNA sequencing analysis. Significantly increased RNA (*p* < 0.05) from ovarian cancer ascites (n = 2) compared to benign peritoneal fluid (n = 3) are listed below as either up-regulated or down-regulated in ascites compared to peritoneal fluids. (DOCX 22 kb)

Additional File10: Next generation RNA sequencing data were plotted for each sample of ovarian cancer ascites (OC) and benign peritoneal fluids (DC). RPKM values for each corresponding gene are indicated in blue and the 30 selected genes for qPCR validation are labeled in red. Solid blue lines indicate linear regression. Both over- and under-expressed genes were selected for validation. (PPTX 318 kb)

Additional File 11: Multivariate discriminant analysis of mRNA and miRNA qPCR data. Linear discriminant functions are listed for ovarian cancer and disease control groups. (DOCX 12 kb)

## Abbreviations

CA125: Cancer antigen 125
EOC: Epithelial ovarian cancer
EV: Extracellular vesicles
FTSEC: Fallopian tube secretory epithelial cell
