# Supplementary material for: Comparison of benign peritoneal fluid- and ovarian cancer ascites-derived extracellular vesicle RNA biomarkers
Source: J Ovarian Res. 2018 Mar 2;11:20. doi: 10.1186/s13048-018-0391-2 (PMC5834862; doi:10.1186/s13048-018-0391-2)
Supplement: Supplementary file 1 — Primer sequences (5′ to 3′) for qPCR validation. (DOCX 13 kb) [file 13048_2018_391_MOESM1_ESM.docx]

**Additional File1. Primer sequences (5’ to 3’) for qPCR validation**

Gene Sense Antisense

*NANOG* *gccaggatggtctcgatctc ggtggctcacgcctgtaaat*

*ZEB2* *aagataggtggcgcgtgttt ctttcggccactccaggaa*

*SPINT2*  *tcccacgctggtactttgac aaccaccacctttgagccaa*

*AZGP1 tgcagggaaggtttggttgt ttggttatctgggctgctgg*

*LGALS7B ggaggtggtcttcaacagca tcgtctgacgcgatgatgag*

*LINC00251 ggtgagcagctgactcagtt ttggagaggaggactagccc*

*LCE1F ctcctgtctcttcctgctgc ctgcagcagtcagagctctg*

*HNF1A-AS1 ctgggtttgagcctcgttct gggattgcaggtgtgatcca*

*OR8D2 ccacagtccatactacccgc aaggaccgccagtgtagttg*

*PSCA tgctgtgctactcctgcaaa tcatccacgcagttcaagct*

*TFF3 ctgtctgcaaaccagtgtgc tcctggagtcaaagcagcag*

*MTRNR2L1 aggacatcccaatggtgcag tgaagtgggccccatttctc*

*LAMA4 ggaagtgcactcgagaacca ttggcgtttttgcttccgag*

*RBP1 aatgtggccttgcgcaaaat tgcctgtcagatcctcctca*

*MMP2 tgatggcatcgctcagatcc ggcctcgtataccgcatcaa*

*CA11 tgttccctgaatccttcggc gaggtgatattgagggcccg*

*GZMA ggtggaagagactcgtgcaa tatagacaccaggcccacga*

*IGFBP1 ggcacaggagacatcaggag agacccagggatcctcttcc*

*MTRNR2L10 ctccgcaaattttaccccgc ctgcggccattgaacgtatg*

*KLK5 caaagtgcttggtgtctggc gtctcgggtaagcatcctcg*

*GPX3 agtatgtccgaccaggtgga aaagttccagcggatgtcgt*

*IL8 ctccaaacctttccacccca ttccttggggtccagacaga*

*SERPING1 ctcctacccagcccactact ttgctgagaaggcgtggtag*

*RNASE7 ctcaagcatgcaactcagcc gcaggctattttgggggtct*

*ODF2L agccaagtggaacctgcaat tcagacaacttggcttcctga*

*MEDAG gcaagggatggacatggtca accacttcatttcctggggg*

*LUM gtggtaccagtggccagtac attccaggaggcaccattgg*

*LYPD3 tctgacctccgcaacaagac ctccctgtctcggagtctga*

*CSTA acggaaaattggaagctgtgc cgtcagctcgtcatccttgt*

*UPK1B accaaaacaacagccctcca ggccagggatagtcagcatc*

*S100A16 gcagtcattgtcctggtgga gatgagcttatccgcagcct*

*SPARC caagaagccctgcctgatga tcttcggtttcctctgcacc*

*GAPDH cccactcctccacctttgac cataccaggaaatgagcttgacaa*

*ACTB* *tttttcctggcacccagcacaat tttttgccgatccacacggagtact*

*B2M* *tgactttgtcacagcccaagata aatgcggcatcttcaaacct*
