# Supplementary material for: Comparison of benign peritoneal fluid- and ovarian cancer ascites-derived extracellular vesicle RNA biomarkers
Source: J Ovarian Res. 2018 Mar 2;11:20. doi: 10.1186/s13048-018-0391-2 (PMC5834862; doi:10.1186/s13048-018-0391-2)
Supplement: Supplementary file 2 — Clinical information from patient ascites samples. (DOCX 13 kb) [file 13048_2018_391_MOESM2_ESM.docx]

**Additional File2. Clinical information from patient ascites samples**

**___________________________________________________________________________________________________________**

**Sample Age Diagnosis FIGO CA125 Primary Response Progression Overall**

**(years) stage (kU/L) chemothx to chemothx free survival survival**

**(regimen x cycle #) (months) (months)**

1 48 LGS IV 1,769 neo-adj C/T x4+2 NR n/a 19.67

2 58 HGS IIIC 1,700 C/T x 6 CR 15.42 15.42

3 54 HGS IIIC n/a C/T x 6 NR 12.3 74.6

4 80 HGS IIIC n/a C/T x 6 CR 10.87 23.94

5 70 HGS n/a 1,485 F/U n/a n/a n/a

6 80 HGS IIIC 1,115 C/T x 6 CR 31 34.7

7 56 HGS n/a 1,369 C/T x 6 CR 23.1 29.55

8 69 HGS n/a 296 C/T x 6 n/a n/a n/a

LGS = low grade serous, HGS = high grade serous, C/T = cisplatin/taxane, NR = no response, CR = complete response, n/a = not available
