# Supplementary material for: Comparison of benign peritoneal fluid- and ovarian cancer ascites-derived extracellular vesicle RNA biomarkers
Source: J Ovarian Res. 2018 Mar 2;11:20. doi: 10.1186/s13048-018-0391-2 (PMC5834862; doi:10.1186/s13048-018-0391-2)
Supplement: Supplementary file 3 — Clinical information from benign peritoneal fluid (PF) sample pathology reports. (DOCX 12 kb) [file 13048_2018_391_MOESM3_ESM.docx]

**Additional File3. Clinical information from benign peritoneal fluid (PF) sample pathology reports.**

| **Sample** | **Peritoneal Washings** | **Right Ovary** | **Left Ovary** | **Right Tube** | **Left Tube** | **Uterus and Cervix** |
| --- | --- | --- | --- | --- | --- | --- |
| 1 | no malignant cells | paratubal cysts | adenomatoid tumor (benign), BRCA2 mutation | n/a | n/a | n/a |
| 2 | no malignant cells | endometrioma | peritubal adhesions | endometriosis | n/a | n/a |
| 3 | no malignant cells | paratubal cysts | paratubal cysts; pelvic mass; leiomyoma | n/a | n/a | n/a |
| 4 | no malignant cells | cystic follicles | corpus luteum, BRCA1 mutation (neoplasm of left breast) | n/a | n/a | n/a |
| 5 | no malignant cells | epidermoid cyst | cortical inclusion cysts | n/a | n/a | n/a |
| 6 | no malignant cells | no change | endometrioma | hydrosalpinx | n/a | n/a |
| 7 | no malignant cells | endometrioma | n/a | n/a | n/a | n/a |
| 8 | no malignant cells | endometriotic cyst | endometriotic cysts | n/a | no change | squamous metaplasia |
| 9 | no malignant cells | cystic follicles, inclusion cysts | cystic follicles and inclusion cysts | paratubal cysts | paratubal cysts, MLH1 mutation | n/a |
| 10 | no malignant cells | mucinous cystadenoma | cortical inclusion cysts | paratubal cysts | paratubal cysts | n/a |
