# Supplementary material for: Comparison of benign peritoneal fluid- and ovarian cancer ascites-derived extracellular vesicle RNA biomarkers
Source: J Ovarian Res. 2018 Mar 2;11:20. doi: 10.1186/s13048-018-0391-2 (PMC5834862; doi:10.1186/s13048-018-0391-2)
Supplement: Supplementary file 4 — Preliminary set of genes with primer sequences for screening based on literature search. Additional genes (MET, EGFR, EPCAM, CLDN3) were quantitated using Qiagen Quantitect Primer Assays. (DOCX 13 kb) [file 13048_2018_391_MOESM4_ESM.docx]

**Additional File4. Preliminary set of genes with primer sequences for screening based on literature search. Additional genes (*MET, EGFR, EPCAM, CLDN3*) were quantitated using Qiagen Quantitect Primer Assays.**

Gene Sense Antisense

*CD24* gacactccccgaagtcttttgt *tcatcaagactactgtggccatattag*

*CD44 gaaaaatggtcgctacagcatct tgtgggcaaggtgctattga*

*MCAM ggcagcacagcccttctg agcgtccgcttctccttgt*

*TNFSF7 cacactctgcaccaacctcact tgcactccaaagaaggtctcatc*

*NRCAM tgattacgtggaagcccttga cacaaccacagatgtccattcat*

*RASAL1 tggatttctcttcttgcgattct tgttggtcccgaaggtcaa*

*SH2D3A ctggccaaccttggtacca caggaagtcgccattttgct*

*CLDN7 ttgccgccttggtagctt tggtagggatcaaagggttataaaa*

*SFN gccctgaacttttccgtcttc aaagtggtcttggccagagaga*

*C8ORF4 agccaccaagccatcatcat gaagtggtagccatggatgga*

*IL23A caaatgatgttccccatatcca cagaactgactgttgtccctgagt*

*SPINT2 ccactccagcgatatgttcaac cggcaaggcccagtga*

*GATA3 ttagagccctgctcgatgct catgatactgctcctgcaaaaatg*

*CST6 tcaagtacttcctgacgatggagat gtcgacgtggtctccagtga*

*PAX8 tcttactctaagcccaacgctttt cctccactgggtctagaaccat*

*KRT8 ctgggatgcagaacatgagtattc gcttgtgaggcccccatag*

*CCNE1 aaagaagatgatgaccgggtttac gagcctctggatggtgcaat*

*MYC cccccaaggtagttatccttaaaaa cgcaacaagtcctcttcagaaa*

*SOX2 tgcgagcgctgcacat gcagcgtgtacttatccttcttca*

*NANOG gccaggatggtctcgatctc ggtggctcacgcctgtaaat*

*TGM2 ttggccccgcctaagg catattttgctcactagcttgggata*

*vimentin ggattcactccctctggttgat gttgataacctgtccatctctagtttca*

*twist1 gcgctgcggaagatcatc gcttgagggtctgaatcttgct*

*zeb2 aagataggtggcgcgtgttt ctttcggccactccaggaa*

*HIF-1a tgaacataaagtctgcaacatgga tgaggttggttactgttggtatcatata*

*zeb1 gcccagttacccacaatcgt tgaccgtagttgagtaggtgtatgc*

*ACTN4 tgtgccccaaaagactatcca caccttgggcggcttgt*

*TMEM158 gctgaaccgtaagcccattg cgctccacaccacgatgac*

*AKT2 gatcctgcggaaggaagtca cgggtgcctggtgttctg*

*FALEC tggatcacgaggtcaggagtt gctcctcttcctcctgtatgttct*

*N-cadherin tgggaatccgacgaatgg gcagatcggaccggatactg*

*P-cadherin gcttactccatccatagccaaga gcctgtgctccggtgaat*

*E-cadherin aaatctgaaagcggctgatactg cggaaccgcttccttcatag*

*MMP-9 ccagtaccgagagaaagcctattt ggtcacgtagcccacttggt*

*MMP-14 caacaaccagaagctgaaggtaga cgtccacctcaatgatgatcac*

*MMP-2 cggcggtcacagctacttc ttcacgctcttcagactttggtt*

*TG2 ctttgacgtctttgcccacat cggtgcgggcacaga*

*VEGFA tcatcacgaagtggtgaagttca tcagggtactcctggaagatgtc*

*POU5F1 tggcgtgatctcagctcact ggctgaggcaggagaatcact*

*CTHRC1 tgtttaaaccaaatgggcagtct gcttgactgtgatgctcctacct*

*IL6 tcatcactggtcttttggagtttg tctgcacagctctggcttgt*

*TGFb gggaaattgagggctttcg gaacccgttgatgtccacttg*

*ITGB1 tcagaattggatttggctcattt tggtgcagttctgttcacttgtg*

*GAPDH cccactcctccacctttgac cataccaggaaatgagcttgacaa*

*ACTB tttttcctggcacccagcacaat tttttgccgatccacacggagtact*
