# Supplementary figures and images for: Comparison of benign peritoneal fluid- and ovarian cancer ascites-derived extracellular vesicle RNA biomarkers
Source: J Ovarian Res. 2018 Mar 2;11:20. doi: 10.1186/s13048-018-0391-2 (PMC5834862; doi:10.1186/s13048-018-0391-2)

## Slide 1
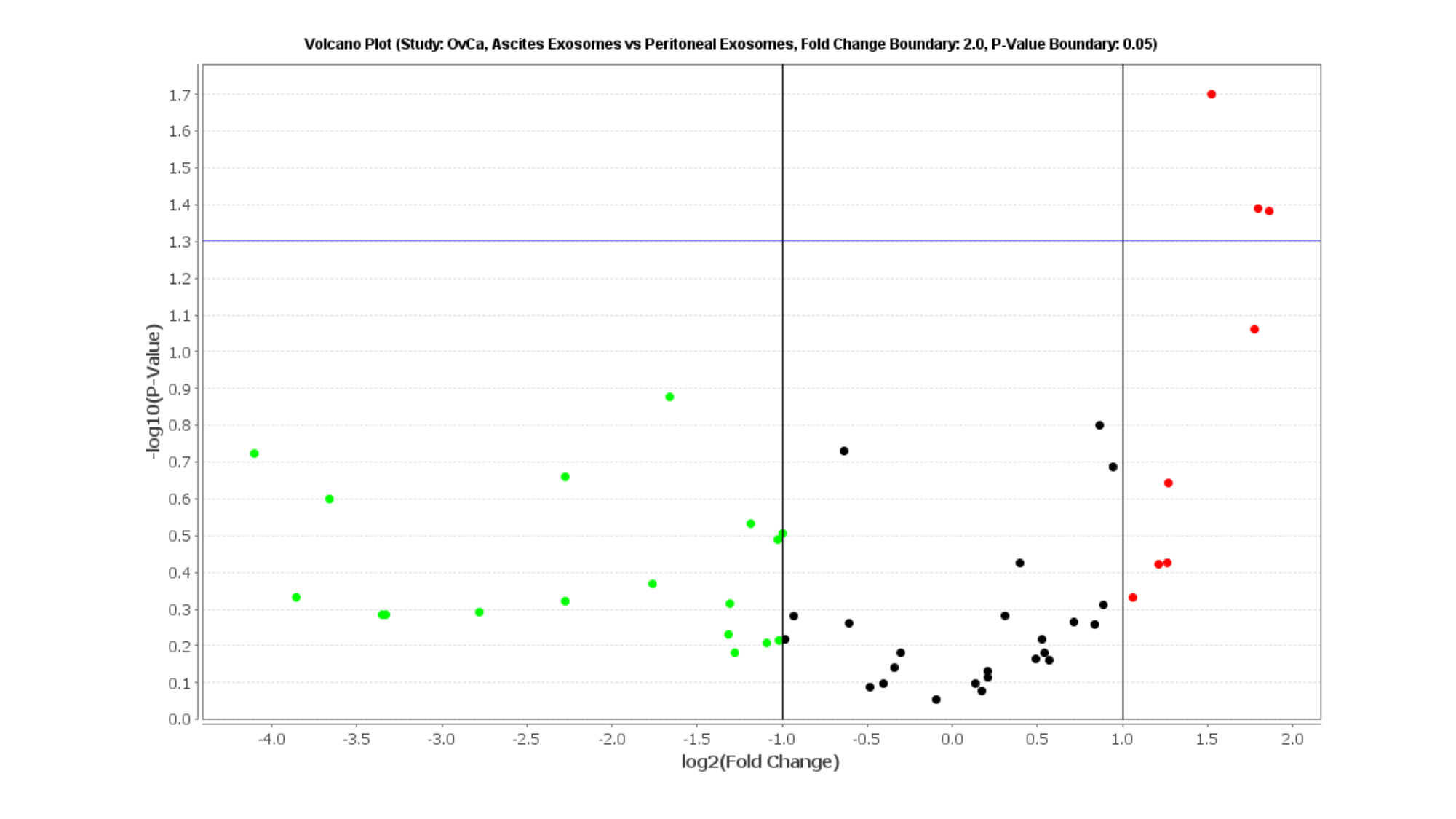

Supplement: Supplementary file 5 — Volcano plot displays p-values vs. fold change of ovarian cancer ascites (n = 8) and peritoneal (n = 2) EVs. Three mRNA (NANOG, SPINT2, ZEB2) show values above the fold change boundary of 2 (2-fold change) and a p-value of 0.05. Plot generated using. Data Assist v3.01 software. (PPTX 58 kb) [file 13048_2018_391_MOESM5_ESM.pptx]

## Slide 1
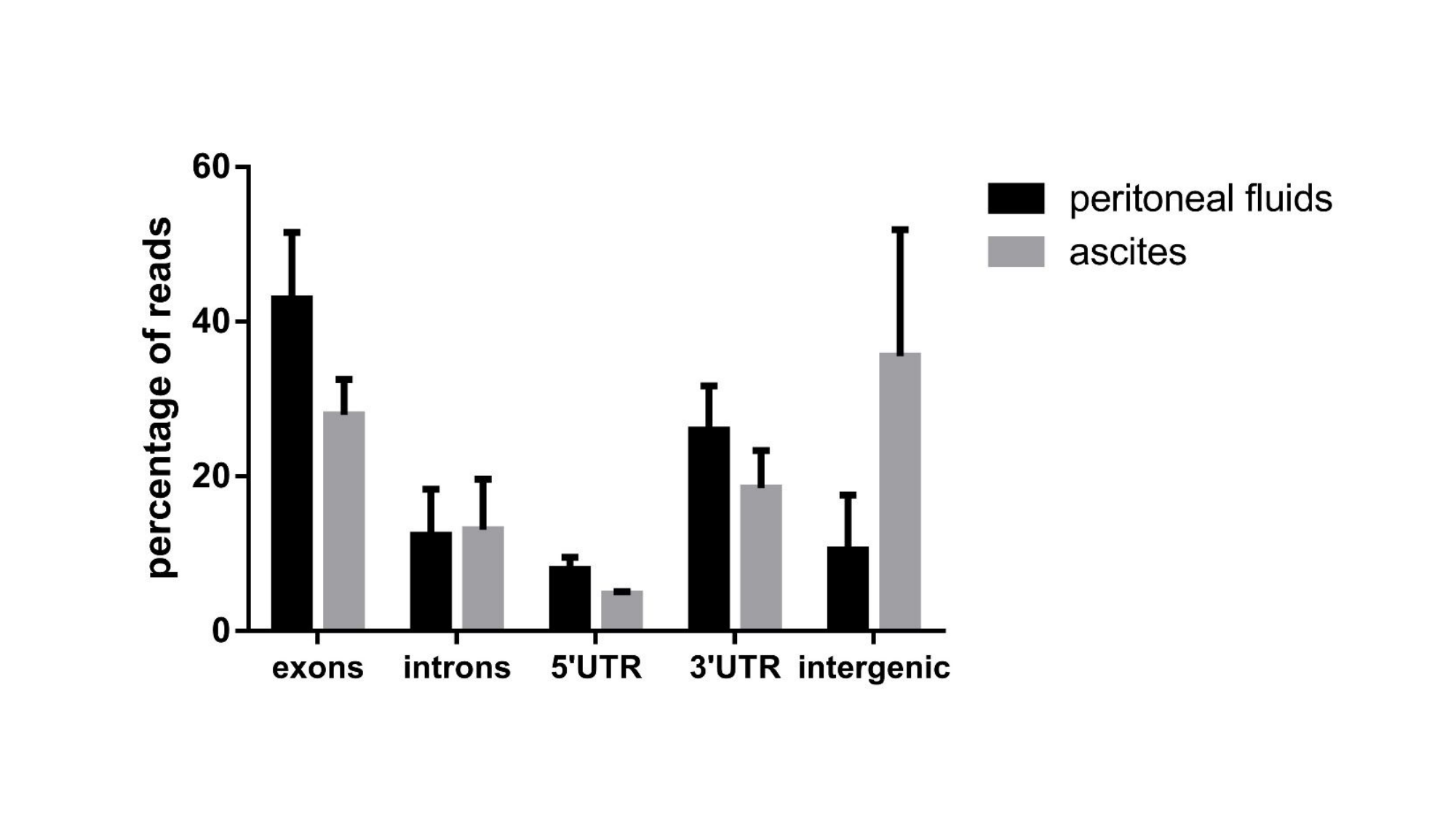

Supplement: Supplementary file 7 — The distribution of EV mRNA sequencing reads mapping to human genome annotations. The percentage of reads (ave. ± SD) overlapping genomic features including exons, introns, UTR, and intergenic regions are shown for peritoneal fluids (n = 3) and ovarian cancer ascites samples (n = 2). (PPTX 115 kb) [file 13048_2018_391_MOESM7_ESM.pptx]

## Slide 1
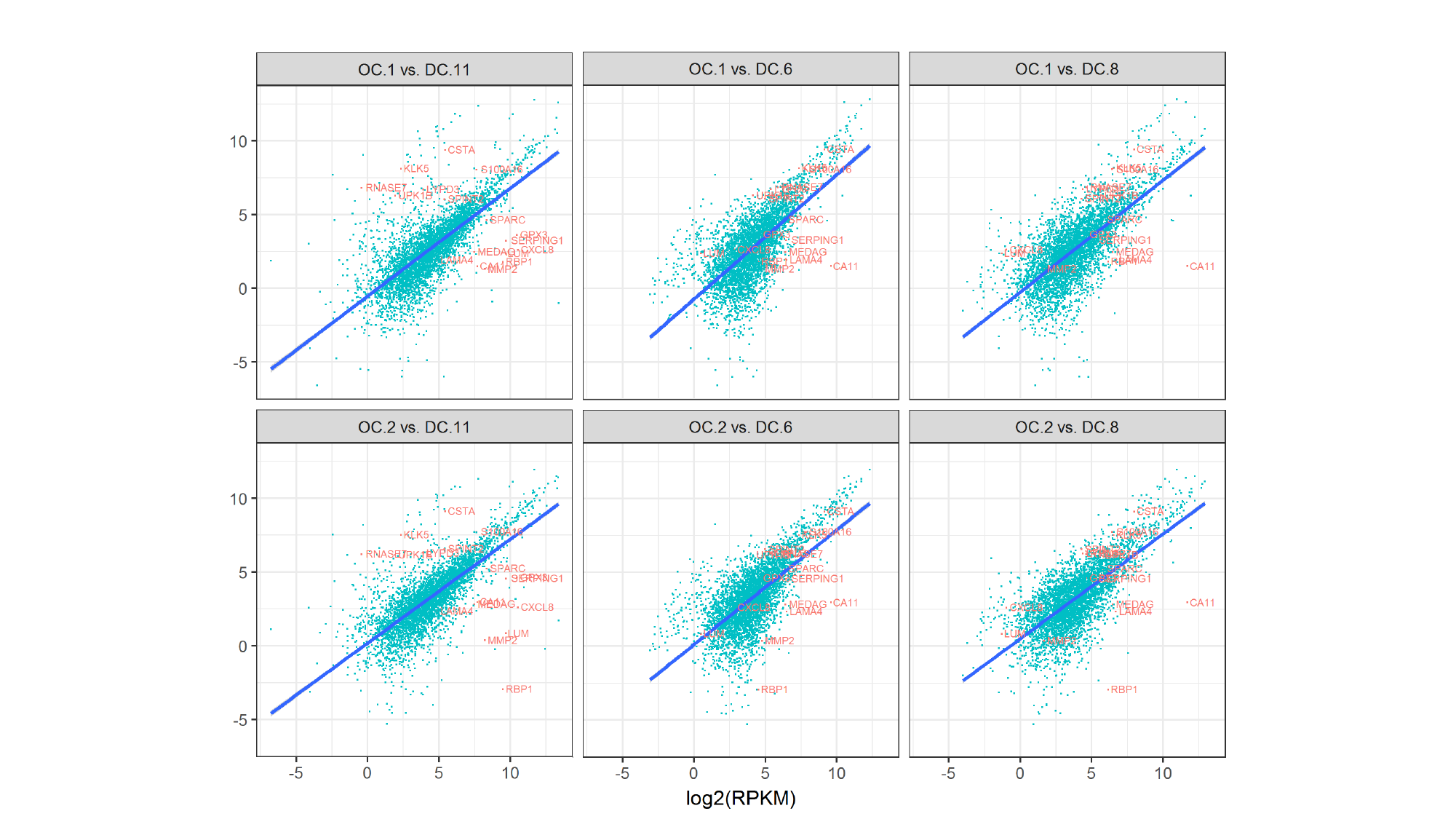

Supplement: Supplementary file 10 — Next generation RNA sequencing data were plotted for each sample of ovarian cancer ascites (OC) and benign peritoneal fluids (DC). RPKM values for each corresponding gene are indicated in blue and the 30 selected genes for qPCR validation are labeled in red. Solid blue lines indicate linear regression. Both over- and under-expressed genes were selected for validation. (PPTX 318 kb) [file 13048_2018_391_MOESM10_ESM.pptx]
