# Supplementary material for: Comparison of benign peritoneal fluid- and ovarian cancer ascites-derived extracellular vesicle RNA biomarkers
Source: J Ovarian Res. 2018 Mar 2;11:20. doi: 10.1186/s13048-018-0391-2 (PMC5834862; doi:10.1186/s13048-018-0391-2)
Supplement: Supplementary file 6 — mRNA sequencing reads and percentage aligned to hg19 assembly. (DOCX 12 kb) [file 13048_2018_391_MOESM6_ESM.docx]

**Additional File6. mRNA sequencing reads and percentage aligned to hg19 assembly.**

|  | **Benign Peritoneal Fluid** | | | **Ovarian Cancer Ascites** | |
| --- | --- | --- | --- | --- | --- |
| **Sample** | **1** | **2** | **3** | **1** | **2** |
| **Total Number of Reads** | 1,917,600 | 2,339,302 | 31,502,877 | 11,784,549 | 6,202,668 |
| **Number of Aligned Reads** | 950,205 | 1,551,165 | 26,839,422 | 10,306,830 | 4,936,241 |
| **Percent of Reads Aligned** | **49.55%** | **66.30%** | **85.19%** | **87.46%** | **79.58%** |
