# Supplementary material for: Comparison of benign peritoneal fluid- and ovarian cancer ascites-derived extracellular vesicle RNA biomarkers
Source: J Ovarian Res. 2018 Mar 2;11:20. doi: 10.1186/s13048-018-0391-2 (PMC5834862; doi:10.1186/s13048-018-0391-2)
Supplement: Supplementary file 8 — Ingenuity Pathway Analysis summary of top diseases and disorders, top canonical pathways, and top molecular and cellular functions. P-values are calculated using the right-tailed Fisher Exact Test and number of molecules are based on Ingenuity Knowledge Base with information contained in Canonical Pathways coming from specific journal articles, review articles, textbooks and HumanCyc. (DOCX 22 kb) [file 13048_2018_391_MOESM8_ESM.docx]

**Additional File 8. Ingenuity Pathway Analysis summary of top diseases and disorders, top canonical pathways, and top molecular and cellular functions. P-values are calculated using the right-tailed Fisher Exact Test and number of molecules are based on Ingenuity Knowledge Base with information contained in Canonical Pathways coming from specific journal articles, review articles, textbooks and HumanCyc.**

| **Top Diseases and Disorders** | **p-value range** | **#Molecules** |
| --- | --- | --- |
| Cancer | 3.11E-02 - 6.91E-09 | 43 |
| Organismal Injury and Abnormalities | 3.11E-02 - 6.91E-09 | 44 |
| Reproductive System Disease | 2.62E-02 - 6.91E-09 | 29 |
|  |  |  |
| **Top Canonical Pathways** | **p-value** | **Overlap** |
| Retinoate Biosynthesis I | 2.60E-03 | 5.90% |
| Retinoate Biosynthesis II | 8.87E-03 | 25.00% |
| Communication between Innate and Adaptive Immune Cells | 1.68E-02 | 2.20% |
|  |  |  |
| **Top Molecular and Cellular Functions** | **p-value range** | **#Molecules** |
| Carbohydrate Metabolism | 1.99E-02 - 7.23E-05 | 7 |
| Small Molecule Biochemistry | 3.07E-02 - 7.23E-05 | 12 |
| Molecular Transport | 3.07E-02 - 7.81E-04 | 12 |
