# Supplementary material for: Comparison of benign peritoneal fluid- and ovarian cancer ascites-derived extracellular vesicle RNA biomarkers
Source: J Ovarian Res. 2018 Mar 2;11:20. doi: 10.1186/s13048-018-0391-2 (PMC5834862; doi:10.1186/s13048-018-0391-2)
Supplement: Supplementary file 9 — Differentially expressed genes from RNA sequencing analysis. Significantly increased RNA (p < 0.05) from ovarian cancer ascites (n = 2) compared to benign peritoneal fluid (n = 3) are listed below as either up-regulated or down-regulated in ascites compared to peritoneal fluids. (DOCX 22 kb) [file 13048_2018_391_MOESM9_ESM.docx]

**Additional File 9. Differentially expressed genes from RNA sequencing analysis. Significantly increased RNA (p < 0.05) from ovarian cancer ascites (n=2) compared to benign peritoneal fluid (n=3) are listed below as either up-regulated or down-regulated in ascites compared to peritoneal fluids.**

| **UP-REGULATED IN ASCITES** | | |
| --- | --- | --- |
| **ID** | **SYMBOL** | **GENE DESCRIPTION** |
| 242 | ALOX12B | arachidonate 12-lipoxygenase, 12R type |
| 100507501 | LOC100507501 | uncharacterized LOC100507501 |
| 563 | AZGP1 | alpha-2-glycoprotein 1, zinc-binding |
| 100506421 | LOC100506421 | uncharacterized LOC100506421 |
| 100288748 | LOC100288748 | uncharacterized LOC100288748 |
| 100287765 | LINC00630 | long intergenic non-protein coding RNA 630 |
| 1041 | CDSN | corneodesmosin |
| 1088 | CEACAM8 | carcinoembryonic antigen-related cell adhesion molecule 8 |
| 653499 | LGALS7B | lectin, galactoside-binding, soluble, 7B |
| 646982 | LINC00598 | long intergenic non-protein coding RNA 598 |
| 646903 | LOC646903 | uncharacterized LOC646903 |
| 643486 | LOC643486 | bromodomain, testis-specific pseudogene |
| 552859 | LINC00251 | long intergenic non-protein coding RNA 251 |
| 440051 | KRTAP5-11 | keratin associated protein 5-11 |
| 3270 | HRC | histidine rich calcium binding protein |
| 401027 | C2orf66 | chromosome 2 open reading frame 66 |
| 353139 | LCE2A | late cornified envelope 2A |
| 353137 | LCE1F | late cornified envelope 1F |
| 353132 | LCE1B | late cornified envelope 1B |
| 3952 | LEP | leptin |
| 339975 | LOC339975 | uncharacterized LOC339975 |
| 339665 | SLC35E4 | solute carrier family 35, member E4 |
| 339298 | LOC339298 | uncharacterized LOC339298 |
| 284656 | EPHA10 | EPH receptor A10 |
| 284486 | THEM5 | thioesterase superfamily member 5 |
| 283460 | HNF1A-AS1 | HNF1A antisense RNA 1 |
| 283160 | OR8D2 | olfactory receptor, family 8, subfamily D, member 2 |
| 219447 | OR5AS1 | olfactory receptor, family 5, subfamily AS, member 1 |
| 200879 | LIPH | lipase, member H |
| 170392 | OIT3 | oncoprotein induced transcript 3 |
| 6582 | SLC22A2 | solute carrier family 22 (organic cation transporter), member 2 |
| 7033 | TFF3 | trefoil factor 3 (intestinal) |
| 7143 | TNR | tenascin R |
| 8000 | PSCA | prostate stem cell antigen |
| 146439 | CCDC64B | coiled-coil domain containing 64B |
| 145241 | ADAM21P1 | ADAM metallopeptidase domain 21 pseudogene 1 |
| 8797 | TNFRSF10A | tumor necrosis factor receptor superfamily, member 10a |
| 133060 | OTOP1 | otopetrin 1 |
| 131177 | FAM3D | family with sequence similarity 3, member D |
| 126410 | CYP4F22 | cytochrome P450, family 4, subfamily F, polypeptide 22 |
| 125965 | COX6B2 | cytochrome c oxidase subunit VIb polypeptide 2 (testis) |
| 121214 | SDR9C7 | short chain dehydrogenase/reductase family 9C, member 7 |
| 84189 | SLITRK6 | SLIT and NTRK-like family, member 6 |
| 80339 | PNPLA3 | patatin-like phospholipase domain containing 3 |
| 420 | ART4 | ADP-ribosyltransferase 4 (Dombrock blood group) |
| 57119 | EPPIN | epididymal peptidase inhibitor |
| 56169 | GSDMC | gasdermin C |
| 100462977 | MTRNR2L1 | MT-RNR2-like 1 |
| 100463488 | MTRNR2L10 | MT-RNR2-like 10 |
| 27076 | LYPD3 | LY6/PLAUR domain containing 3 |
| 84659 | RNASE7 | ribonuclease, RNase A family, 7 |
| 25818 | KLK5 | kallikrein-related peptidase 5 |
| 57489 | ODF2L | outer dense fiber of sperm tails 2-like |
| 1475 | CSTA | cystatin A (stefin A) |
| 5108 | PCM1 | pericentriolar material 1 |
| 84935 | MEDAG | mesenteric estrogen-dependent adipogenesis |
| 710 | SERPING1 | serpin peptidase inhibitor, clade G (C1 inhibitor), member 1 |
| 3910 | LAMA4 | laminin, alpha 4 |
| 2878 | GPX3 | glutathione peroxidase 3 (plasma) |
| 5947 | RBP1 | retinol binding protein 1, cellular |
| 4060 | LUM | lumican |
| 4313 | MMP2 | matrix metallopeptidase 2 (gelatinase A, 72kDa gelatinase, 72kDa type IV collagenase) |
| 3576 | IL8 | interleukin 8 |
| 770 | CA11 | carbonic anhydrase XI |
| 158787 | RIBC1 | RIB43A domain with coiled-coils 1 |
| 85001 | MGC16275 | uncharacterized protein MGC16275 |
| 89866 | SEC16B | SEC16 homolog B (S. cerevisiae) |
| 91179 | SCARF2 | scavenger receptor class F, member 2 |
| 91461 | PKDCC | protein kinase domain containing, cytoplasmic |
| 92070 | CTBP1-AS2 | CTBP1 antisense RNA 2 (head to head) |
| 11080 | DNAJB4 | DnaJ (Hsp40) homolog, subfamily B, member 4 |
| 10486 | CAP2 | CAP, adenylate cyclase-associated protein, 2 (yeast) |
| 9506 | PAGE4 | P antigen family, member 4 (prostate associated) |
| 128869 | PIGU | phosphatidylinositol glycan anchor biosynthesis, class U |
| 9194 | SLC16A7 | solute carrier family 16 (monocarboxylate transporter), member 7 |
| 8929 | PHOX2B | paired-like homeobox 2b |
| 139818 | DOCK11 | dedicator of cytokinesis 11 |
| 140738 | TMEM37 | transmembrane protein 37 |
| 8613 | PPAP2B | phosphatidic acid phosphatase type 2B |
| 8418 | CMAHP | cytidine monophospho-N-acetylneuraminic acid hydroxylase, pseudogene |
| 29948 | OSGIN1 | oxidative stress induced growth inhibitor 1 |
| 164312 | LRRN4 | leucine rich repeat neuronal 4 |
| 165186 | FAM179A | family with sequence similarity 179, member A |
| 6004 | RGS16 | regulator of G-protein signaling 16 |
| 56475 | RPRM | reprimo, TP53 dependent G2 arrest mediator candidate |
| 201514 | ZNF584 | zinc finger protein 584 |
| 27152 | INTU | inturned planar cell polarity protein |
| 255231 | MCOLN2 | mucolipin 2 |
| 5104 | SERPINA5 | serpin peptidase inhibitor, clade A (alpha-1 antiproteinase, antitrypsin), member 5 |
| 5016 | OVGP1 | oviductal glycoprotein 1, 120kDa |
| 4867 | NPHP1 | nephronophthisis 1 (juvenile) |
| 4838 | NODAL | nodal growth differentiation factor |
| 285905 | INTS4L1 | integrator complex subunit 4-like 1 |
| 4482 | MSRA | methionine sulfoxide reductase A |
| 29091 | STXBP6 | syntaxin binding protein 6 (amisyn) |
| 57577 | KIAA1407 | KIAA1407 |
| 340074 | LOC340074 | uncharacterized LOC340074 |
| 55070 | DET1 | de-etiolated homolog 1 (Arabidopsis) |
| 64926 | RASAL3 | RAS protein activator like 3 |
| 3560 | IL2RB | interleukin 2 receptor, beta |
| 400793 | C1orf226 | chromosome 1 open reading frame 226 |
| 3484 | IGFBP1 | insulin-like growth factor binding protein 1 |
| 3250 | HPR | haptoglobin-related protein |
| 3001 | GZMA | granzyme A (granzyme 1, cytotoxic T-lymphocyte-associated serine esterase 3) |
| 2921 | CXCL3 | chemokine (C-X-C motif) ligand 3 |
| 80031 | SEMA6D | sema domain, transmembrane domain (TM), and cytoplasmic domain, (semaphorin) 6D |
| 2861 | GPR37 | G protein-coupled receptor 37 (endothelin receptor type B-like) |
| 80321 | CEP70 | centrosomal protein 70kDa |
| 25907 | TMEM158 | transmembrane protein 158 (gene/pseudogene) |
| 722 | C4BPA | complement component 4 binding protein, alpha |
| 55592 | GOLGA2P5 | golgin A2 pseudogene 5 |
| 55617 | TASP1 | taspase, threonine aspartase, 1 |
| 29083 | GTPBP8 | GTP-binding protein 8 (putative) |
| 60625 | DHX35 | DEAH (Asp-Glu-Ala-His) box polypeptide 35 |
| **DOWN-REGULATED IN ASCITES** | | |
| **ID** | **SYMBOL** | **GENE DESCRIPTION** |
| 5108 | PCM1 | pericentriolar material 1 |
| 84935 | MEDAG | mesenteric estrogen-dependent adipogenesis |
| 710 | SERPING1 | serpin peptidase inhibitor, clade G (C1 inhibitor), member 1 |
| 3910 | LAMA4 | laminin, alpha 4 |
| 2878 | GPX3 | glutathione peroxidase 3 (plasma) |
| 5947 | RBP1 | retinol binding protein 1, cellular |
| 4060 | LUM | lumican |
| 4313 | MMP2 | matrix metallopeptidase 2 (gelatinase A, 72kDa gelatinase, 72kDa type IV collagenase) |
| 3576 | IL8 | interleukin 8 |
| 770 | CA11 | carbonic anhydrase XI |
| 158787 | RIBC1 | RIB43A domain with coiled-coils 1 |
| 85001 | MGC16275 | uncharacterized protein MGC16275 |
| 89866 | SEC16B | SEC16 homolog B (S. cerevisiae) |
| 91179 | SCARF2 | scavenger receptor class F, member 2 |
| 91461 | PKDCC | protein kinase domain containing, cytoplasmic |
| 92070 | CTBP1-AS2 | CTBP1 antisense RNA 2 (head to head) |
| 11080 | DNAJB4 | DnaJ (Hsp40) homolog, subfamily B, member 4 |
| 10486 | CAP2 | CAP, adenylate cyclase-associated protein, 2 (yeast) |
| 9506 | PAGE4 | P antigen family, member 4 (prostate associated) |
| 128869 | PIGU | phosphatidylinositol glycan anchor biosynthesis, class U |
| 9194 | SLC16A7 | solute carrier family 16 (monocarboxylate transporter), member 7 |
| 8929 | PHOX2B | paired-like homeobox 2b |
| 139818 | DOCK11 | dedicator of cytokinesis 11 |
| 140738 | TMEM37 | transmembrane protein 37 |
| 8613 | PPAP2B | phosphatidic acid phosphatase type 2B |
| 8418 | CMAHP | cytidine monophospho-N-acetylneuraminic acid hydroxylase, pseudogene |
| 29948 | OSGIN1 | oxidative stress induced growth inhibitor 1 |
| 164312 | LRRN4 | leucine rich repeat neuronal 4 |
| 165186 | FAM179A | family with sequence similarity 179, member A |
| 6004 | RGS16 | regulator of G-protein signaling 16 |
| 56475 | RPRM | reprimo, TP53 dependent G2 arrest mediator candidate |
| 201514 | ZNF584 | zinc finger protein 584 |
| 27152 | INTU | inturned planar cell polarity protein |
| 255231 | MCOLN2 | mucolipin 2 |
| 5104 | SERPINA5 | serpin peptidase inhibitor, clade A (alpha-1 antiproteinase, antitrypsin), member 5 |
| 5016 | OVGP1 | oviductal glycoprotein 1, 120kDa |
| 4867 | NPHP1 | nephronophthisis 1 (juvenile) |
| 4838 | NODAL | nodal growth differentiation factor |
| 285905 | INTS4L1 | integrator complex subunit 4-like 1 |
| 4482 | MSRA | methionine sulfoxide reductase A |
| 29091 | STXBP6 | syntaxin binding protein 6 (amisyn) |
| 57577 | KIAA1407 | KIAA1407 |
| 340074 | LOC340074 | uncharacterized LOC340074 |
| 55070 | DET1 | de-etiolated homolog 1 (Arabidopsis) |
| 64926 | RASAL3 | RAS protein activator like 3 |
| 3560 | IL2RB | interleukin 2 receptor, beta |
| 400793 | C1orf226 | chromosome 1 open reading frame 226 |
| 3484 | IGFBP1 | insulin-like growth factor binding protein 1 |
| 3250 | HPR | haptoglobin-related protein |
| 3001 | GZMA | granzyme A (granzyme 1, cytotoxic T-lymphocyte-associated serine esterase 3) |
| 2921 | CXCL3 | chemokine (C-X-C motif) ligand 3 |
| 80031 | SEMA6D | sema domain, transmembrane domain (TM), and cytoplasmic domain, (semaphorin) 6D |
| 2861 | GPR37 | G protein-coupled receptor 37 (endothelin receptor type B-like) |
| 80321 | CEP70 | centrosomal protein 70kDa |
| 25907 | TMEM158 | transmembrane protein 158 (gene/pseudogene) |
| 722 | C4BPA | complement component 4 binding protein, alpha |
| 55592 | GOLGA2P5 | golgin A2 pseudogene 5 |
| 55617 | TASP1 | taspase, threonine aspartase, 1 |
| 29083 | GTPBP8 | GTP-binding protein 8 (putative) |
| 60625 | DHX35 | DEAH (Asp-Glu-Ala-His) box polypeptide 35 |
