# Supplementary material for: Comparison of benign peritoneal fluid- and ovarian cancer ascites-derived extracellular vesicle RNA biomarkers
Source: J Ovarian Res. 2018 Mar 2;11:20. doi: 10.1186/s13048-018-0391-2 (PMC5834862; doi:10.1186/s13048-018-0391-2)
Supplement: Supplementary file 11 — Multivariate discriminant analysis of mRNA and miRNA qPCR data. Linear discriminant functions are listed for ovarian cancer and disease control groups. (DOCX 12 kb) [file 13048_2018_391_MOESM11_ESM.docx]

**Additional File 11. Multivariate discriminant analysis of mRNA and miRNA qPCR data. Linear discriminant functions are listed for ovarian cancer and disease control groups.**

|  | Ovarian Cancer | Disease Control |  |
| --- | --- | --- | --- |
| Constant | -1130.7 | -1065.8 |  |
| *LAMA4* | 8.7 | 9.6 |  |
| *CA11* | 1.9 | 0.7 |  |
| *MEDAG* | -3.8 | -3.4 |  |
| *NANOG* | -8.0 | -8.3 |  |
| *SPINT2* | 31.7 | 33.2 |  |
| *let7b* | -60.9 | -63.8 |  |
| *miR23b* | 31.9 | 26.4 |  |
| *miR29a* | 65.6 | 69.5 |  |
|  |  |  |  |
